# The mechanisms of social support and psychological resilience on the comprehensive wellbeing of healthcare professionals

**DOI:** 10.3389/fpubh.2026.1793167

**Published:** 2026-04-13

**Authors:** Sheng Li, Yuting Li, Qing Jia, Fei Wang, Wenjie Liu, Guoai Yao, Jinyu Wang

**Affiliations:** 1 Lanzhou NO.2 People's Hospital, Lanzhou, Gansu, China; 2 The First Clinical Medical College, Gansu University of Chinese Medicine, Lanzhou, Gansu, China; 3 Lanzhou Pulmonary Hospital, Lanzhou, Gansu, China; 4 School of Public Health, Gansu University of Chinese Medicine, Lanzhou, Gansu; 5 School of Basic Medical Sciences, Lanzhou University, Lanzhou, Gansu, China

**Keywords:** general wellbeing, healthcare professionals, hospital, psychological resilience, social support

## Abstract

**Objective:**

To explore the influence paths and relationship effects of social support and psychological resilience on the comprehensive wellbeing status of healthcare workers.

**Methods:**

The social support rating scale, psychological resilience scale and self-administered comprehensive wellbeing questionnaire were used to investigate 696 healthcare workers in a hospital. Structural equation modeling was constructed using Amos 26.0 software to analyse the influence paths.

**Results:**

The total score of comprehensive wellbeing was positively correlated with the total score of the Social Support Rating Scale and the total score of the Psychological Resilience Scale, and the total score of the Psychological Resilience Scale was positively correlated with the total score of the Social Support Rating Scale. Psychological elasticity played a mediating role between social support and comprehensive wellbeing.

**Conclusion:**

The overall general wellbeing of healthcare workers in this hospital is average, social support and psychological elasticity affect the general wellbeing of healthcare workers, and social support can indirectly affect an individual's general wellbeing through psychological elasticity.

## 1 Introduction

Happiness, as a core subject of long-standing human exploration, encompasses both factual judgments about objective living conditions and value judgments concerning subjective individual experiences (1). With the development of positive psychology, research on wellbeing has gradually evolved into two classic paradigms: subjective wellbeing and psychological wellbeing. The former emphasizes emotional experiences and life satisfaction, focusing on whether an individual feels happy; the latter originates from the traditions of eudaimonism and humanism, emphasizing self-actualization and optimal psychological functioning. However, in real-world occupational contexts, an individual's state of wellbeing is often influenced by both internal psychological resources and external environmental conditions, making a single-dimensional approach to wellbeing inadequate for fully reflecting overall quality of life. The concept of “comprehensive wellbeing” proposed by Miao Yuanjiang integrates subjective experiences with objective situational factors, including dimensions such as emotional state, life satisfaction, career development, income level, and social recognition (2), thereby providing a more context-sensitive theoretical perspective for studying the wellbeing of occupational groups.

Although several well-established measurement tools for wellbeing exist—such as the Subjective Wellbeing Scale, the Psychological Wellbeing Scale, and the WHO-5 Wellbeing Index—these instruments tend to focus on single dimensions such as emotional experience or psychological functioning, offering limited reflection on objective factors closely related to the occupational context (e.g., career development opportunities, income and benefits, professional identity). For specific occupational groups like healthcare professionals, the experience of wellbeing derives not only from individual emotional states but is also closely linked to factors such as the work environment, career progression, and organizational recognition. Therefore, relying solely on general wellbeing scales may inadequately capture the overall wellbeing of healthcare professionals within their specific occupational context. Grounded in the theoretical framework of comprehensive wellbeing and considering the actual working conditions of healthcare professionals in primary medical institutions, this study developed a comprehensive wellbeing measurement tool based on existing theories and literature. This tool aims to systematically evaluate the holistic wellbeing experiences of this occupational group across areas such as income and benefits, career planning, professional identity, and job satisfaction.

In the field of occupational health research, the stress-buffering theory posits (3) that social support can mitigate the adverse effects of high-stress situations on individuals' mental health and wellbeing. The core mechanism lies in the ability of social support—through the provision of emotional and instrumental resources—to moderate the relationship between stressful events and psychological outcomes. Among healthcare professionals, a typical high-stress occupational group, long-term heavy workloads, emotional labor, and persistent psychological strain constitute significant occupational risk factors (4). Against this backdrop, social support, as a key contextual resource, may serve a protective role in high-stress environments and be closely associated with individuals' overall state of wellbeing.

Furthermore, from the perspective of the Job Demands-Resources (JD-R) model (5), the high job demands inherent in healthcare professions may activate an exhaustion pathway, whereas job resources facilitate positive psychological outcomes through a motivational pathway. Existing JD-R research has predominantly focused on outcome variables such as work engagement or job satisfaction, with relatively limited exploration from the integrated perspective of comprehensive wellbeing as a positive outcome dimension. Therefore, incorporating comprehensive wellbeing into the JD-R framework not only aligns with the “resource moderation” logic of the stress-buffering theory but also expands the explanatory scope of the JD-R model in the realm of positive psychological outcomes. Based on this theoretical foundation, it can be inferred that social support, as a crucial job resource, is likely positively associated with the comprehensive wellbeing of healthcare professionals.

Conservation of Resources (COR) Theory provides a dynamic explanation for this resource mechanism (6). According to COR theory, individuals strive to obtain, retain, and protect valued resources, and resource acquisition can trigger “resource gain spirals,” leading to sustained positive psychological outcomes (7). In high-stress occupational contexts, social support, as an external resource, may activate and enhance an individual's internal psychological resources, creating a process of resource accumulation. Psychological resilience, as a core personal resource, reflects an individual's ability to maintain adaptation and functional stability in the face of adversity. When social support fosters the development of psychological resilience, individuals are more likely to enter a resource gain cycle, thereby maintaining or enhancing their overall level of wellbeing.

Meanwhile, Positive Organizational Behavior (POB) emphasizes the critical role of developable positive psychological resources in organizational settings, which include resilience, self-efficacy, and hope, among others (8). Psychological resilience, as a key component of psychological capital, is more readily strengthened within supportive organizational environments (9). Integrating the reciprocal determinism perspective from social cognitive theory (10), it can be understood that social support—as an environmental factor—enhances individuals' cognitive appraisal and emotion regulation capabilities, thereby promoting the formation and consolidation of psychological resilience, which in turn is associated with higher levels of comprehensive wellbeing.

In summary, within the high-demand context of healthcare professions, an integrated pathway of “job resources → personal resources → comprehensive positive outcomes” can be constructed: social support may not only be directly associated with comprehensive wellbeing but may also, by strengthening psychological resilience as a personal resource, serve both buffering and motivational functions in stressful situations, thereby promoting an enhanced overall state of wellbeing. This integrated framework incorporates the protective mechanism of the stress-buffering theory, the motivational pathway of the JD-R model, the resource gain logic of COR theory, and the core perspective of POB on psychological capital development, thereby providing a systematic theoretical foundation for explaining the formation mechanism of comprehensive wellbeing among healthcare professionals.

## 2 Subjects and methods

### 2.1 Study population

This cross-sectional cluster survey was conducted from November 10 to 15, 2024, involving 745 frontline clinical healthcare workers at a provincial-level hospital. Exclusion criteria were as follows: (1) questionnaire completion time exceeding 40 min or less than 3 min; (2) History of mental illness; (3) Non-frontline clinical staff (including administrative and support personnel). This study was approved by the Medical Ethics Committee of Lanzhou Pulmonary Hospital (Approval No.: 202311101), and all participants provided informed consent.

### 2.2 Research methodology

A questionnaire survey was conducted from November 10 to 15, 2024, targeting frontline medical staff at a provincial-level hospital. The questionnaire was developed and distributed via the “Quanxunxing” platform. A pilot survey was conducted prior to the formal survey, and revisions were made based on the results. All participants were fully informed of the study objectives and completion requirements. A total of 745 questionnaires were distributed, with 738 returned. Among these, 696 were valid, yielding an effective response rate of 94.31%. The questionnaire primarily comprised the following four sections:

#### 2.2.1 General information

Includes gender, ethnicity, department, years of service, educational background, professional title, average monthly income, and desired income.

#### 2.2.2 Comprehensive wellbeing questionnaire

The Comprehensive Wellbeing Scale used in this study is a measurement tool independently developed by the research team based on existing theoretical frameworks. The scale development process strictly followed classical scale construction procedures, adhering to the construct measurement development paradigm proposed by Churchill (11), and implemented systematic steps including “construct definition, item generation, item purification, reliability, and validity testing.” Simultaneously, referring to Hinkin's operational guidelines for questionnaire development (12), content validity and construct validity were ensured through expert evaluation and factor analysis procedures. During the item design and revision stage, optimizations were made based on Robert F. DeVellis's recommendations regarding item clarity, unidimensionality, and psychometric robustness (13).

During the theoretical construction phase, this study, grounded in theories related to subjective wellbeing, job satisfaction, and organizational identification, and considering the actual working context of healthcare professionals in primary medical institutions, established a four-dimensional structural model. This model includes four latent dimensions: income and Benefits, Career Planning, Professional Identity, and Job Satisfaction. An initial item pool was generated through literature review and research team discussions. After screening items and revising their semantics based on expert consultation, a final formal scale comprising 45 items across the four dimensions was formed. All items were rated on a 5-point Likert scale, with higher scores indicating a higher level of comprehensive wellbeing. The raw total score for each dimension is the sum of all item scores within that dimension. Due to the varying number of items across dimensions, the raw total scores are not directly comparable. To facilitate cross-dimensional comparisons and inclusion in structural equation modeling analysis, this study applied standardized transformation to the raw total scores of each dimension. The overall Cronbach's α coefficient for the questionnaire was 0.831; the Cronbach's α coefficients for the respective dimensions were 0.975, 0.975, 0.973, and 0.901.

#### 2.2.3 Social Support Rating Scale (SSRS)

The Chinese version of the Social Support Rating Scale (SSRS), revised by Xiao Shuiyuan, was employed (14). This scale comprises three dimensions: subjective support (4 items), objective support (3 items), and utilization of support (3 items). A higher score indicates a higher level of perceived social support. In this study, the scale demonstrated a Cronbach's α coefficient of 0.789.

#### 2.2.4 Connor-Davidson Resilience Scale (CD-RISC)

The Chinese version of the Psychological Resilience Scale, revised by Yu et al., was used. It comprises three dimensions—resilience, strength, and optimism—with 25 items (15). In this study, the Cronbach's α coefficient for this scale was 0.857.

### 2.3 Statistical analysis

Data were exported using Excel 2021 and analyzed with SPSS 27.0. The Shapiro-Wilk test assessed normality of quantitative data. Since questionnaire dimension scores did not follow normal distribution, quantitative data are presented as M (P25, P75) and categorical data as [*n* (%)]. Before conducting correlation analysis, a normality test was performed on the main study variables. The results indicated that some variables did not fully meet the assumption of normal distribution. Furthermore, as the data were derived from a 5-point Likert scale, they were considered ordinal. Consequently, Spearman's rank correlation coefficient was used in the bivariate correlation analysis phase to examine the relationships among social support, psychological resilience, and comprehensive wellbeing. Subsequently, to further test the research hypotheses and the structural relationships among the latent variables, Structural Equation Modeling (SEM) was employed for path analysis. Specifically, AMOS 26.0 software was used to construct the structural equation model to analyze the pathways through which social support and psychological resilience affect comprehensive wellbeing. A *p*-value of less than 0.05 was considered statistically significant.

## 3 Results

### 3.1 Common method bias

This study employed self-report data collection, which may introduce common method bias. To address this, the Harman one-factor test was applied for diagnosis. Results indicate that the variance explained by the first common factor extracted without rotation was 30.51%, below the critical threshold of 40.00%. This suggests that common method bias does not pose a significant threat to data quality, allowing for subsequent analysis.

### 3.2 Basic information of healthcare personnel

A total of 696 valid questionnaires were collected, including 666 female respondents (95.70%) and 30 male respondents (4.30%); 683 respondents (98.10%) were Han Chinese. Nurses outnumbered physicians, with 417 nurses (59.91%). Departmental distribution showed higher representation in internal medicine, surgery, obstetrics, and pediatrics, totaling 578 respondents (83.00%). Years of service predominantly fell within the 6–10 year and 11–19 year ranges, with 275 (39.50%) and 273 (39.20%) respondents, respectively. Bachelor's degrees constituted the majority of educational backgrounds, held by 585 (84.10%) respondents. Intermediate professional titles were the most common technical designation, held by 370 (53.20%) respondents. See Table 1 for details.

**Table 1:** Basic information of medical staff.

Characteristic	Grouping	Frequency	Composition ratio
Gender	Female	666	95.70%
	Male	30	4.30%
Ethnicity	Han Chinese	683	98.10%
	Minority ethnic group	13	1.90%
Occupation	Physician	279	40.09%
	Nurse	417	59.91%
Department	Internal medicine, surgery, obstetrics, gynecology, pediatrics	578	83.00%
	Emergency, outpatient	51	7.30%
	Operating room	33	4.70%
	Supply room	34	4.90%
Years of Service	≤ 5years	82	11.80%
	6~10years	275	39.50%
	11~19years	273	39.20%
	≥20years	66	9.50%
Education	Vocational school	2	0.30%
	College	101	14.50%
	Undergraduate	585	84.10%
	Graduate or higher	8	1.10%
Professional Title	Senior	27	3.90%
	Associate senior	211	30.30%
	Intermediate	370	53.20%
	Junior	88	12.60%

### 3.3 Basic income status of healthcare personnel

This survey found that 9.48% of respondents had a monthly average income ≤ 3,000 yuan, while 52.44% earned between 3,001 and 6,000 yuan monthly. Regarding desired monthly income, the highest proportion of healthcare workers (422 individuals, 60.63%) aspired to earn 9,001 yuan or more. Regarding desired salary increases, the number of respondents seeking a 1–2 grade increase was relatively close, at 267 (38.36%), and 250 (35.92%), respectively. Only 11 individuals (1.58%) expressed a desire to maintain their current income or accept a reduction. See Table 2 for details.

**Table 2:** Basic income and benefits of healthcare personnel.

Related factors	Grouping	Frequency	Composition ratio
Monthly average income (yuan)	≤ 3,000	66	9.48%
	3,001~6,000	365	52.44%
	6,001~9,000	144	20.69%
	≥9,001	121	17.39%
Monthly desired income (yuan)	≤ 3,000	2	0.29%
	3,001~6,000	66	9.48%
	6,001~9,000	206	29.60%
	≥9,001	422	60.63%
Compensation gap (yuan)	Maintain current or decrease	11	1.58%
	Increase by 1 grade	267	38.36%
	Increase by 2 grades	250	35.92%
	Increase by 3 grades or more	168	24.14%

### 3.4 Current status of healthcare workers' social support, psychological resilience, and overall wellbeing

The standardized scores for each dimension of the Comprehensive Wellbeing Questionnaire were as follows: the mean raw item score for the Income and Benefits dimension was 3.25 (median: 2.88, 3.36), for the Career Planning dimension was 3.00 (median: 3.00, 3.82), for the Professional Identity dimension was 3.69 (median: 3.00, 4.08), and for the Job Satisfaction dimension was 3.46 (median: 3.00, 4.00). The overall mean raw item score for the Comprehensive Wellbeing Questionnaire was 67.11 (median: 60.44, 78.67). The total scores and dimensional scores for social support and psychological resilience (see Table 3).

**Table 3:** Current status of healthcare workers' social support, psychological resilience, and overall wellbeing.

Project	Grouping	M (P_25, _ P_75_) score
Social support scale	Objective support dimension total score	8.00 (5.00, 8.00)
	Subjective support dimension total score	25.00 (21.00, 28.00)
	Support utilization dimension total score	7.50 (6.00, 9.00)
	Social support scale total score	39.00 (33.00, 43.00)
Psychological resilience scale	Resilience dimension total score	26.00 (22.00, 37.00)
	Power dimension total score	19.00 (15.00, 24.00)
	Optimism dimension total score	8.00 (6.00, 11.00)
	Psychological resilience scale total score	54.00 (45.00, 71.00)
Comprehensive wellbeing questionnaire	Income and benefits dimension total score	11.56 (10.22, 12.89)
	Career planning dimension total score	14.67 (14.67, 18.67)
	Career identity dimension total score	21.33 (17.33, 23.56)
	Job satisfaction dimension total score	20.00 (17.33, 23.11)
	Comprehensive wellbeing questionnaire total score	67.11 (60.44, 78.67)

### 3.5 Correlation analysis of healthcare workers' social support, psychological resilience, and overall wellbeing

Spearman correlation analysis revealed that healthcare workers' total social support score was positively correlated with their overall wellbeing score (*r* = 0.278, *P* < 0.001) and with their total psychological resilience scale score (*r* = 0.327, *P* < 0.001). The total score on the psychological resilience scale was positively correlated with the total score on the overall wellbeing scale (*r* = 0.393, *P* < 0.001). See Table 4 for details.

**Table 4:** Correlation analysis of nursing staff's social support, psychological resilience, and overall wellbeing questionnaire scores (*r*).

Project	A	B	C	D	E	F	G	H	I
Total Social Support Score	1.000^***^								
Objective Support Dimension	0.584^***^	1.000^***^							
Subjective Support Dimension	0.915^***^	0.363^***^	1.000^***^						
Support Utilization Dimension	0.653^***^	0.329^***^	0.397^***^	1.000^***^					
Total Psychological Resilience Score	0.327^***^	0.242^***^	0.249^***^	0.315^***^	1.000^***^				
Resilience Dimension	0.317^***^	0.231^***^	0.241^***^	0.308^***^	0.962^***^	1.000^***^			
Power Dimension	0.327^***^	0.249^***^	0.253^***^	0.299^***^	0.956^***^	0.872^***^	1.000^***^		
Optimism Dimension	0.282^***^	0.200^***^	0.220^***^	0.256^***^	0.865^***^	0.762^***^	0.822^***^	1.000^***^	
Total Comprehensive Wellbeing Score	0.278^***^	0.128^***^	0.246^***^	0.254^***^	0.393^***^	0.366^***^	0.416^***^	0.333^***^	1.000^***^

A = Total Social Support Score, B = Objective Support Dimension, C = Subjective Support Dimension, D = Support Utilization Dimension, E = Total Psychological Resilience Score, F = Resilience Dimension, G = Power Dimension, H = Optimism Dimension, I = Total Comprehensive Wellbeing Score; ^***^ *P* < 0.001.

### 3.6 Analysis of factors influencing healthcare workers' comprehensive wellbeing: social support and psychological resilience

#### 3.6.1 Structural equation modeling for mediating effect analysis

**Figure 1:**
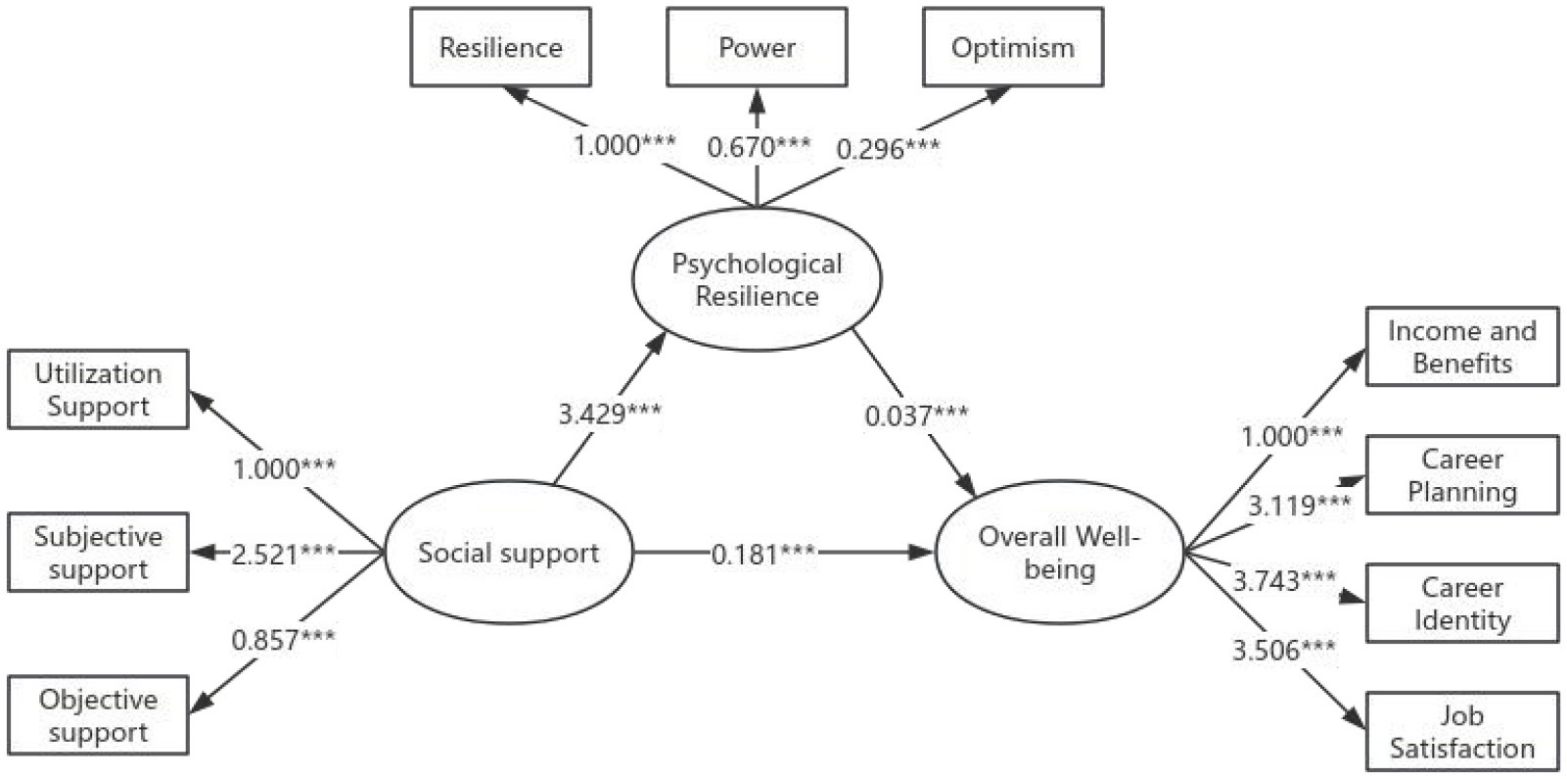
Analysis of the mediation model. ^***^P < 0.001.

A structural equation model was constructed with overall wellbeing as the dependent variable, total social support as the independent variable, and psychological resilience as the mediating variable. Standardized path coefficients were calculated. Model fit was assessed, with results indicating all fit indices met reference standards, confirming good model fit and adequate alignment between the mediating model and sample data. Details are presented in Table 5 and Figure 1.

**Table 5:** Mediation model fit results.

Evaluation indicators	Grouping	Model indicators	Reference standards
Absolute fit indicators	*X^2^/df*	3.32	<5
	GFI	0.971	>0.9
	AGFI	0.950	>0.9
	RMSEA	0.058	<0.08
Incremental fit indicators	CFI	0.984	>0.9
	NFI	0.977	>0.9
	TLI	0.977	>0.9
	IFI	0.984	>0.9
Parsimonious fit indices	PNFI	0.695	>0.5
	PCFI	0.700	>0.5

#### 3.6.2 Mediating path analysis based on structural equation modeling

According to the Fornell-Larcker criterion proposed by Claes Fornell and David F. Larcker (16), an Average Variance Extracted (AVE) value of not less than 0.50 is generally recommended to indicate adequate convergent validity of a latent variable. However, in applied research, when the Composite Reliability (CR) reaches an acceptable level (generally considered ≥0.60), the measurement model can still be deemed to have basically acceptable convergent validity even if the AVE is slightly below 0.50. Relevant methodological studies have pointed out that in social science survey research, when the CR is greater than 0.60 and all measurement indicator loadings are statistically significant, an AVE slightly below 0.50 does not necessarily negate the convergent validity of the latent variable. In this study, the CR for the latent variable of social support was 0.664, which meets the minimum acceptable standard commonly used in exploratory research (0.60), indicating that the internal consistency of this latent variable remains within an acceptable range. Although its AVE value was 0.397, below the recommended threshold of 0.50, all observed indicator factor loadings for this latent variable reached statistical significance, and the overall structural model fit indices were within acceptable ranges. Therefore, this study contends that the convergent validity of this latent variable remains reasonably acceptable under the conditions of the current sample.

The analysis results indicate that all path coefficients are statistically significant (P <0.001). Specifically, the measurement model path analysis revealed standardized path coefficients between observed and latent variables ranging from 0.594 to 0.988, indicating strong explanatory power of each variable for the overall model. The structural model path analysis showed standardized path coefficients between latent variables ranging from 0.201 to 0.402. See Table 6 for details.

**Table 6:** Path coefficients of the mediated model.

Path	Unstd	Std	SE	*T*-Value	*P*	SMC	CR	AVE
Psychological resilience → social support	3.429	0.402	0.441	7.768	<0.001			
Overall wellbeing → psychological resilience	0.037	0.356	0.005	7.700	<0.001			
Overall wellbeing → social support	0.181	0.201	0.047	3.873	<0.001			
Support utilization → social support	1.000	0.634				0.402	0.664	0.397
Perceived support → social support	2.521	0.661	0.240	10.496	<0.001	0.437		
Objective support → social support	0.857	0.594	0.083	10.275	<0.001	0.353		
Resilience → psychological resilience	1.000	0.923				0.852	0.948	0.860
Power → psychological resilience	0.670	0.988	0.013	51.968	<0.001	0.976		
Optimism → psychological resilience	0.296	0.867	0.008	36.667	<0.001	0.752		
Income and benefits → overall wellbeing	1.000	0.560				0.314	0.891	0.679
Career planning → overall wellbeing	3.119	0.864	0.196	15.880	<0.001	0.746		
Career identity → overall wellbeing	3.743	0.911	0.230	16.277	<0.001	0.830		
Job satisfaction → overall wellbeing	3.506	0.909	0.216	16.264	<0.001	0.826		

#### 3.6.3 Testing mediating effects using structural equation modeling

To assess the significance of mediating effects, a bias-corrected nonparametric percentile bootstrap method was employed. Results indicate an indirect effect value of 0.128 (95% CI: 0.082, 0.191), which does not include zero. This confirms the indirect effect, demonstrating that psychological resilience partially mediates the relationship between healthcare workers' social support and overall wellbeing. Calculations based on the proportion of effect size reveal that psychological resilience accounts for 41.42% of the effect. See Table 7 for details.

**Table 7:** Mediation model effect values.

Project	Effect size	Boot LLCI	Boot ULCI	*P*
Indirect effect	0.128	0.082	0.191	0.006
Total effect	0.309	0.213	0.418	0.009
Effect ratio	0.414	0.236	0.719	0.007

## 4 Discussion

### 4.1 Main findings

This study explored the relationships among social support, psychological resilience, and the comprehensive wellbeing of healthcare professionals. The results demonstrated significant positive correlations between social support and comprehensive wellbeing, as well as between psychological resilience and comprehensive wellbeing. Structural equation modeling further revealed that psychological resilience plays a partial mediating role in the relationship between social support and comprehensive wellbeing. These findings suggest that within high-stress occupational environments, both external social support resources and internal individual psychological resources are closely associated with the overall wellbeing of healthcare professionals. From a resource integration perspective, this study elucidates the pathway linking “social support → psychological resilience → comprehensive wellbeing,” providing new empirical evidence for understanding the formation of positive psychological states among healthcare professionals.

### 4.2 Comparison with existing research

The finding of a significant positive correlation between social support and comprehensive wellbeing is largely consistent with previous research on the relationship between social support and mental health. Prior studies have indicated that social support can alleviate an individual's psychological burden in stressful situations by providing emotional support and practical assistance, thereby correlating with better mental health outcomes (3). The similar finding within the healthcare professional cohort in this study further underscores the significant role of social support in high-stress occupational environments (17). Moreover, the significant positive correlation between psychological resilience and comprehensive wellbeing aligns with previous conclusions regarding resilience's role in promoting adaptive capacity and positive psychological states (18, 19). Psychological resilience is recognized as a crucial resource for maintaining psychological stability and functional adaptation when facing stress or adversity; consequently, individuals with higher resilience are more likely to sustain positive life experiences within complex work environments (20).

Further mediation analysis indicated that psychological resilience plays a partial mediating role between social support and comprehensive wellbeing. This result corresponds with the view presented in some existing research that “social resources promote the development of psychological resources” (21), suggesting that social support may not only be directly associated with individual wellbeing but may also exert an indirect influence by enhancing internal psychological resources.

### 4.3 Theoretical explanations

The findings of this study can be interpreted from several theoretical perspectives. First, from the viewpoint of the stress-buffering hypothesis, social support can mitigate the adverse effects of stress on mental health when individuals confront high-stress situations (3). Healthcare professionals commonly face substantial workloads and emotional pressure in their work; thus, support resources from the organization, colleagues, and family may help alleviate the negative impacts of occupational stress, thereby correlating with more positive psychological experiences. Second, through the lens of the Job Demands–Resources (JD-R) Model, the high job demands inherent in healthcare professions may trigger a health impairment pathway, whereas job resources may foster positive psychological outcomes via a motivational pathway [22]. Within this framework, social support can be conceptualized as a significant job resource, while comprehensive wellbeing represents a more integrated positive outcome variable. By incorporating comprehensive wellbeing into the JD-R framework, this study extends, to some extent, the model's applicability in occupational health research. Furthermore, the Conservation of Resources (COR) Theory offers additional insight into the relationship between social support and psychological resilience. This theory posits that individuals strive to acquire and maintain valued resources, and resource acquisition can initiate a “resource gain spiral” (7). In this study, social support, as an external resource, may facilitate the development of internal psychological resources—such as psychological resilience—thus initiating a process of resource accumulation that is associated with higher levels of comprehensive wellbeing.

### 4.4 Practical implications

From a practical perspective, the findings of this study suggest that in high-stress occupational environments, attention to healthcare professionals' social support resources and psychological resource development may hold significant importance. First, healthcare institutions can enhance the perceived level of social support among medical staff by fostering a supportive organizational environment, promoting teamwork, and strengthening interactive support among colleagues. Second, providing enhanced training and career development support during professional growth and organizational management processes could also contribute to improving employees' positive psychological experiences. Additionally, focusing on the development of individual psychological resources is similarly of potential significance. For instance, implementing stress management training, mental health education, or resilience-building programs may help strengthen healthcare professionals' ability to cope with occupational stress, thereby correlating with more positive psychological experiences. Although this study cannot directly prove the effectiveness of such interventions, the relevant findings provide reference directions for future exploration of intervention strategies aimed at enhancing the mental health and wellbeing of healthcare professionals.

### 4.5 Study limitations and future research directions

While this study sheds light on the relationships between social support, psychological resilience, and the comprehensive wellbeing of healthcare professionals, several limitations should be considered when interpreting the findings. First, the cross-sectional design employed here, with all data collected at a single time point, precludes definitive causal inferences between the variables. Future research could adopt longitudinal or experimental designs to explore potential dynamic relationships among these variables. Second, the sample was drawn from a single healthcare institution, limiting the geographical and organizational diversity, which may affect the generalizability of the results. Additionally, imbalances in the sample composition regarding gender and professional categories might also influence the outcomes. Future studies should validate these findings using multi-regional or multicenter samples. Third, the Comprehensive Wellbeing Scale used in this study was developed by the research team based on relevant theoretical frameworks. Although its development followed established scale construction methodologies and preliminary tests of reliability and validity were conducted, further validation across diverse samples and contexts is necessary to enhance its measurement stability and generalizability. Furthermore, as the data primarily relied on self-report questionnaires, there is a potential for common method bias. Future research could mitigate this by incorporating multi-source data or multi-wave data collection approaches. Finally, while this study focused on social support and psychological resilience, the formation of comprehensive wellbeing among healthcare professionals is likely influenced by multiple individual and organizational factors. Future studies could integrate a broader range of variables within a more comprehensive theoretical framework to deepen the understanding of the mechanisms underlying wellbeing in healthcare professionals.

### 4.6 Research conclusion

This study investigated the relationships between social support, psychological resilience, and comprehensive wellbeing among healthcare professionals from a stress perspective. The results indicate that both social support and psychological resilience are positively correlated with comprehensive wellbeing. Furthermore, psychological resilience plays a partial mediating role between social support and comprehensive wellbeing, suggesting that external social resources may influence wellbeing both directly and indirectly through internal psychological resources. These findings provide empirical support for an integrated framework linking contextual resources, personal resources, and positive outcomes in high-demand occupational environments. By adopting comprehensive wellbeing as an outcome variable, this study also contributes to extending existing theoretical perspectives—including the stress-buffering hypothesis, the Job Demands-Resources (JD-R) model, and Conservation of Resources (COR) theory—within the context of healthcare professionals. Overall, the results highlight the potential importance of supportive work environments and the development of psychological resilience for the overall wellbeing of healthcare professionals. Future research should employ longitudinal or multi-center designs to further clarify the relationships identified in this study.

## Data Availability

The original contributions presented in the study are included in the article/Supplementary material, further inquiries can be directed to the corresponding authors.

## References

[1] Timm I Giurgiu M Ebner-Priemer U Reichert M. The within-subject association of physical behavior and affective well-being in everyday life: a systematic literature review. Sports Med. (2024) 54:1667–705. doi: 10.1007/s40279-024-02016-1 38705972 PMC11239742

[2] Yuanjiang M Haobin C. Research on the Happiness of Chinese People. Beijing: Beijing Normal University Press (2020). p. 448.

[3] Cohen S Wills TA. Stress, social support, and the buffering hypothesis. Psychol Bull. (1985) 98:310–57 3901065

[4] Qi X Zang CP. Current status and influencing factors of overall happiness among healthcare staff in cadre health care units of a tertiary general hospital in Beijing. Chin J Clini Healthcare. (2020) 23:260–3. doi: 10.3969/J.issn.1672-6790.2020.02.030

[5] Bakker AB Demerouti E. The job demands-resources model: state of the art. J Managerial Psychol. (2007) 22:309–28. doi: 10.1108/02683940710733115

[6] Hobfoll SE. Conservation of resources. a new attempt at conceptualizing stress. Am Psychol. (1989) 44:513–24. doi: 10.1037/0003-066X.44.3.513 2648906

[7] Hobfoll SE. The influence of culture, community, and the nested-self in the stress process: advancing conservation of resources theory. Appl Psychol. (2001) 50:337–421. doi: 10.1111/1464-0597.00062

[8] Luthans F. Positive organizational behavior: developing and managing psychological strengths. Acad Manag Perspect. (2002) 16:57–72. doi: 10.5465/ame.2002.6640181

[9] Luthans F Youssef-Morgan CM Avolio BJ. Psychological Capital: Developing the Human Competitive Edge. Oxford: Oxford University Press (2006). doi: 10.1093/acprof:oso/9780195187526.001.0001

[10] Bandura A. Social foundations of thought and action: a social cognitive theory. J Appl Psychol. (1986) 12:169. doi: 10.2307/258004

[11] Churchill GA Jr. A paradigm for developing better measures of marketing constructs. J Mark Res. (1979) 16:64–73. doi: 10.1177/002224377901600110

[12] Hinkin TR. A brief tutorial on the development of measures for use in survey questionnaires. Organ Res Methods. (1998) 1:104–21. doi: 10.1177/109442819800100106

[13] Devellis RF. Scale Development: Theory and Applications (Ed.p.1-113). Thousand Oaks, CA: SAGE Publications (2003).

[14] Xiao SY. Theoretical foundation and application studies of the Social Support Rating Scale. J Clinic Psychiatry. (1994) 98–100.

[15] Yu XN Lau JT Mak WW Zhang J Lui WW Zhang J. Factor structure and psychometric properties of the connor-davidson resilience scale among Chinese adolescents. Compr Psychiatry. (2011) 52:218–24. doi: 10.1016/j.comppsych.2010.05.010 21295229

[16] Fornell C Larcker DF. Evaluating structural equation models with unobservable variables and measurement error. J Mark Res. (1981) 24:337–46.

[17] Ozbay F Johnson DC Dimoulas E Morgan CA Charney D Southwick S. Social support and resilience to stress: from neurobiology to clinical practice. Psychiatry. (2007) 4:35–40. 20806028 PMC2921311

[18] Masten AS. Ordinary magic. resilience processes in development. Am Psychol. (2001) 56:227–38. doi: 10.1037/0003-066X.56.3.227 11315249

[19] Bonanno GA. Loss, trauma, and human resilience: have we underestimated the human capacity to thrive after extremely aversive events? Am Psychol. (2004) 59:20–8. doi: 10.1037/0003-066X.59.1.20 14736317

[20] Shatté A Reivich K. The Resilience Factor: 7 Keys to Finding Your Inner Strength and Overcoming Life's Hurdles. New York, NY: Broadway Books (2002).

[21] Demerouti E Bakker AB Nachreiner F Schaufeli WB. The job demands–resources model of burnout. J Appl Psychol. (2001) ā86:499–512. doi: 10.1037/0021-9010.86.3.499 11419809

