# Supplementary material for: The mechanisms of social support and psychological resilience on the comprehensive wellbeing of healthcare professionals
Source: Front Public Health. 2026 Apr 13;14:1793167. doi: 10.3389/fpubh.2026.1793167 (PMC13111297; doi:10.3389/fpubh.2026.1793167)
Supplement: Supplementary file 1 [file Supplementary_file_1.docx]

**Internet Consent Form**

Dear Participants:

Hello! We sincerely invite you to learn about a research study titled "A Study on the Combined Effects of Social Support and Psychological Resilience on the Comprehensive Well-Being of Healthcare Workers from a Stress Perspective." This research aims to explore the combined impact of social support and psychological resilience on the comprehensive well-being of healthcare workers, as well as the underlying mechanisms, in order to provide a scientific basis for enhancing their mental health and optimizing hospital management strategies. Your participation is crucial for ensuring the transparency and ethical compliance of this study.

**I. Purpose and Background of the Study**

This study is a cross-sectional survey designed to analyze the relationship between social support, psychological resilience, and comprehensive well-being among healthcare workers. Data will be collected through questionnaire surveys to examine the pathways through which social support and psychological resilience influence well-being, with a particular focus on the mediating role of psychological resilience. The findings are expected to provide evidence-based references for hospitals and related institutions in developing mental health support measures, improving work environments, and enhancing the job satisfaction and psychological well-being of healthcare workers.**Ⅱ. Questionnaire Content and Form**

This questionnaire is filled out online, and all questions are designed based on the research purpose, aiming to obtain real and objective data. You can complete the questionnaire at any time and place through computers, cell phones, or other terminal devices.

**Ⅲ.Data Confidentiality and Privacy Protection**

We promise to keep your personal information and answers to the questionnaire strictly confidential. Your name, contact information, IP address, and other sensitive information will not be recorded or disclosed.

All data will be used only for the purpose of this study and will be anonymized at the end of the study. We will not use your data for any other purpose or disclose it to third parties.

We will take the necessary technical and administrative measures to ensure the security and integrity of the data and to prevent unauthorized access, alteration, or disclosure of the data.

**IV. Voluntary Participation and Withdrawal**

Participation in this online survey is completely voluntary, and you have the right to choose whether to participate and withdraw at any time.

Your withdrawal will not affect any of your rights and interests, nor will it have a negative impact on this study. You can stop completing the questionnaire at any time without providing any reason or explanation.

**V. Contact and Feedback**

If you have any questions or suggestions about this study, please contact us via the following methods:

Email: Wangjiny@lzu.edu.cn; y18893489441@163.com

We will answer your questions and provide necessary assistance as soon as possible.

**VI. Other Notes**

Please fill in the questionnaire according to your real situation and feelings to ensure the accuracy and reliability of the data.

When filling out the questionnaire, please pay attention to the protection of your privacy and information security to avoid the disclosure of sensitive personal information.

If you encounter any problems or difficulties in the process of filling out the questionnaire, please feel free to contact us, and we will provide you with the necessary support and assistance.

**Participant Acknowledgment:**

☑ I have read and understood the content of the above informed consent form.

☑ I agree to participate in this online questionnaire and authorize the researcher to process my data according to the above requirements.

Date:__

(Note: Please check the appropriate box to confirm informed consent and participation.)

We sincerely thank you for taking the time to participate in this study. Each of your responses contributes valuable insights toward enhancing the mental health and occupational well-being of healthcare workers, carrying positive implications for both public health and society.

Participants in the survey fill out the questionnaire voluntarily in accordance with the requirements of the aforementioned“informed consent form" and may withdraw at any time during the process of filling out the questionnaire.
